# Adolescents' views on oral health care and promotion in Norway: everyday practices, recommendations, and future visions

**DOI:** 10.3389/froh.2024.1290652

**Published:** 2024-04-09

**Authors:** Marikken Høiseth, Arefe Jasbi

**Affiliations:** ^1^ Department of Health Research, SINTEF Digital, SINTEF, Trondheim, Norway; ^2^ Department of Design, Faculty of Architecture and Design, Norwegian University of Science and Technology, Trondheim, Norway

**Keywords:** adolescents, oral health promotion, social inequalities, qualitative methods, oral health practices, recommendations, visions, adolescent perspectives

## Abstract

**Introduction:**

In this study, we aimed to understand adolescents' perspectives on oral health care and promotion. Our research was conducted in the context of Norway's oral health care system, where societal factors like income and education influence health disparities. Despite free public dental care for all residents younger than 19 years, challenges persist in promoting oral health among adolescents, a group whose oral health behavior and literacy remain largely unexplored.

**Materials and methods:**

A thematic analysis of an anonymized dataset from 80 adolescents aged 12–20 years was conducted.

**Results:**

Five central themes were recognized: (1) Feeling fresh vs. feeling indifferent: A broad spectrum of attitudes; (2) Bridging gaps, building habits: Collaborative efforts in oral care; (3) “Create good experiences at the dentist so people come back again”; (4) Requested qualities in oral health promoting solutions; (5) Reminder tools for everyday use. Taken together, these themes highlight adolescents' oral health practices and resources, recommendations for dental clinics, and visions for future oral health promotion.

**Discussion:**

Based on the results, the discussion highlights a need for tailored oral health promotion and ideas to reach adolescents in meaningful and effective ways. Reflections on the theme of social inequalities are provided.

## 1 Introduction

Although studies have documented that oral health has generally improved in the European population during the last decades, a recent systematic review and meta-analysis revealed that tooth decay and other oral health issues remain a common burden during adolescence (1). Our context is Norway, where all residents younger than 19 years receive dental care free of charge through the Public Dental Service (PDS) (2). The exception is orthodontic treatment (3). Families usually get financial aid for orthodontic fixed appliances but often pay part of the cost. Youth aged 19–24 also get discounted public dental care. County municipalities manage oral health promotion, prevention, and dental services for children and prioritized groups (3). However, to what extent adolescents in Norway engage in recommended oral health-promotive behavior is, to a large degree, unknown.

Adolescents' oral health behavior and oral health literacy are generally understudied aspects (4, 5). Additionally, structural factors impacting oral health in childhood and adolescence are not well-defined (6), presenting an important area for further enquiry. A study conducted by Høvik et al. (7) emphasized the need for a more nuanced, targeted approach to improve adolescent oral health in Norway. It highlighted several challenges: the broad individual range in caries lesions rates; the need for specialized promotion, prevention, and treatment for high-risk 12-year-olds; and the opportunity for innovative, adolescent-focused promotive and preventative solutions (7).

Adolescence can be understood as a critical transition phase between childhood dependency on caregivers and the expectations of self-determination and responsibility in adulthood (8). This period is marked by significant socioemotional, cognitive, and physical changes, making adolescents particularly susceptible to various risk factors. Adolescents are typically expected to take greater responsibility for their own health, and their health choices must be seen in connection to well-being, lifestyle, health behaviors, and habits (9, 10). In addition, they also encounter new liberties, such as increased access to money and the ability to make consumer choices, adding complexity to this life stage. As adolescents grapple with forming their identities and a sense of belonging, many exhibit a tendency to distance themselves from established norms and question authority (11). This combination of factors can make understanding the consequences of choices particularly challenging. In the field of oral health, factors that contribute to the risk of tooth decay in adolescents go beyond specific deviant risk-taking behaviors, like drug dependencies (7). General contributors include a lack of attention to oral hygiene and poor dietary habits (7). Other factors that contribute to this risk are a history of tooth decay in primary teeth, being male, and either belonging to a family with low socioeconomic status or being part of a foreign-born family (7). Various studies have established significant associations between lower socioeconomic status indicators and higher rates of caries lesions experience among adolescents (12, 13).

Few oral health promotion interventions targeting adolescents have been developed, and even fewer have involved children in research and design processes. While the use of co-creation for service innovation is increasing in the overall health and care sector (14), there is a lack of evidence on both the application and understanding of design in the oral health field (15). Worldwide, the public dental health service largely seems to lack skills and experiences in co-creating interventions, as only a small proportion of systematically reviewed projects appear to have involved patients or the general public (12). This gap leads us to assume that the best ways to promote oral health in adolescents are yet to be identified. Despite improvements in child participation over the last few decades, most oral health research is conducted *on* children, rather than *with* them (16). In the field of oral health, there is a growing recognition of the need for co-creative strategies in developing and implementing services aimed at improving oral health literacy and practices (17). Co-creative strategies are rooted in the idea that a range of stakeholders, like patients, caregivers, and healthcare professionals, collectively possess a comprehensive view of the challenges and motivations affecting oral health. By actively involving these groups in the creation of new or improved services, we can understand and address barriers that contribute to social and health inequalities (18). Furthermore, co-creative approaches can encourage a sense of empowerment and ownership among participants, enhancing the effectiveness of interventions. Hence, such approaches help amplify diverse voices and empower participants, making them active agents in shaping successful and equitable oral health interventions.

We are interested in the perspectives that adolescents have on oral health care and promotion in a Norwegian context. The study background is an ongoing research and innovation project, #Care4YoungTeeth<3, involving the co-creation of oral health products, digital information, and communication tools, and new service touchpoints, with the overall aim of improving oral health of adolescents in Norway. #Care4YoungTeeth<3 is funded by The Research Council of Norway from 2021 to 2025 (project number 320362). The opportunity for children and youth to be fully and directly involved in decisions that impact their lives is a core principle supported by the United Nations Convention on the Rights of the Child (19). However, the viewpoints of children and young people are often either not collected or overlooked in the development of services. Yet, their perspectives offer valuable insights that can improve the quality of local services aimed at this age group. #Care4YoungTeeth<3 has been inspired by the sociology of childhood, which has made a key contribution in framing of children as social agents with agency, competencies, and the ability to participate (20, 21). This framework emphasizes the importance of listening to children's stories and lived experiences, confirming that their perspectives, social connections, and varied cultures deserve scholarly attention in their own right (20). In line with the sociology of childhood and research on child health and wellbeing (22), and human-centered design principles, situating adolescents as health actors and crucial contributors to the success of oral health promotion practice implies specific investigation into their personal accounts and experiences.

#Care4YoungTeeth<3 is led by SINTEF Digital in collaboration with nine official research partners representing PDS, Norwegian, and international research institutions with combined expertise in oral health, design, and co-creative processes, industry actors producing dental products and solutions for digital storytelling, and finally, a non-profit knowledge center called Changefactory (CF) (23). CF is founded on the idea that children and young people possess important knowledge about public services they are in and make use of, like schools, kindergartens, support services, the police, and the legal system. By collecting experiences and advice from children, CF seeks to position children and young people in shaping and improving public services. This study draws on an anonymized dataset provided by CF on the topic of oral health.

CF gathers insights from children about various public systems through annual surveys (23, 24). This information is summarized and shared in reports, films, and books. Young people with direct experience in these systems are invited to disseminate this knowledge to professionals and students in relevant fields. CF actively includes children in vulnerable situations, those who challenge societal norms, and those with limited trust in adults in their surveys. Additionally, young people aged 13–21 serve as professionals, or “pros” (25), in the initiative, presenting insights, offering further advice, and participating in various activities. All “pros” are invited to participate in CF summer camps.

Summer camp is a yearly event arranged by CF to gather children and youth to work on selected topics and to have a good time with activities like songwriting, swimming, crafting, volleyball, and playing games. As a partner in #Care4YoungTeeth<3, CF introduced oral health as one of the central topics to gather adolescents' input on, entitled the “*tooth assignment”*.

CF uses a method for gathering data that builds on the action research approach known as Participatory Learning and Action (26). The tooth assignment followed CF's standard procedure for assignments in summer camps, with participants rotating through different stations in smaller groups, each with a specific activity or focus, and two CF adults overseeing each station. CF adults are trained in conducting group discussions with children and young people, and writing verbatim reports. The participants are divided into small groups of to facilitate discussion. The size of each group is intentionally kept small to ensure that everyone has an opportunity to speak (24).

## 2 Materials and methods

### 2.1 Research design

The study was guided by the research question: Which perspectives do adolescents have on oral health care and promotion in a Norwegian context? The authors thematically analyzed an anonymized dataset provided by CF. The CF workshop involved 80 adolescents attending two summer camps held in June 2021 in a centrally located town in Norway. Prior to the summer camp, the first author facilitated the CF team including adolescent professionals to co-design the following five questions referred to as the tooth assignment:

1. The dentist and you may have agreed on specific measures for better oral care. Who or what can assist you in remembering to maintain this?
2. What steps do you take or need to take for proper oral care (e.g., brushing, flossing, mouthwash)?
3. What factors influence your willingness or ability to visit the dentist?
4. What do you think is important to know before visiting the dentist?
5. What factors make you feel safe during a dental appointment?

### 2.2 Participants, procedure, and data collection

The participants were 57 girls, 21 boys, and 2 non-binary individuals. The average age was 16.8 years, ranging from 11 years to 20 years. Other demographic data such as race and ethnicity were not collected.

An employee from CF, who was also involved in #Care4YoungTeeth<3, was present at both summer camps to oversee the activity. A brief introduction outlining the purpose of the tooth assignment was given to all participants. The assignment followed the standard procedure for activities, with participants rotating through different stations in smaller groups of 4–5 individuals, and two CF adults present at each station. One adult posed the questions to the group, while the other made verbatim written documentation. CF provided an anonymized 29-page document featuring transcripts of adolescents' statements in response to the tooth assignment. Most of the statements were written in Norwegian, however some statements were in English. The transcripts contained full sentences and direct quotes.

As a gesture of appreciation, a dental care package consisting of a toothbrush, a flossholder, and a toothpaste was distributed to all participants.

### 2.3 Ethical considerations

Participation in CF's summer camp requires that written consent is obtained from the individual participants, or in the case of minors under 16, from both the child and their caregivers. For participants living in institutions, separate written permission is procured from the institution they are part of (24). All involved parties receive detailed information about the camp, including a list of contacts for any questions or concerns they might have. CF seeks to make this information easily understandable to ensure that participants are well-informed about the activities they are signing up for (24).

The project's research process has been approved by Norwegian Agency for Shared Services in Education and Research (Sikt), ref. number 346466.

### 2.4 Data analysis

The authors conducted a thematic analysis on the dataset using an inductive approach, following the methodology outlined by Braun and Clarke (27). The analysis involved several steps: initially, getting familiarized with the data before generating preliminary codes. The CF team had identified some key topics, which were presented and discussed with the #Care4YoungTeeth<3 project team in the fall of 2021. Subsequently, the authors searched for emerging themes, reviewed potential themes for coherence and relevance, and then defined and named the final themes. NVivo software was used to code the transcripts effectively. This process was initially overseen by the first author, who took responsibility for the early stages of analysis and categorization. As part of the first stage of the analysis, a draft of proposed themes along with methodological descriptions was shared orally and in writing with the project team, to establish trustworthiness (28). Selected quotes that were originally in Norwegian were translated into English to support the analysis and for presentation. Eventually, the second author joined to review, validate, and confirm the findings.

## 3 Results

The results comprise five central themes highlighting adolescents' oral health practices and resources, recommendations for dental clinics and visions for future oral health promotion. These are: (1) Feeling fresh vs. feeling indifferent: A broad spectrum of attitudes; (2) Bridging gaps, building habits: Collaborative efforts in oral care; (3) “Create good experiences at the dentist so people come back again”; (4) Requested qualities in oral health promoting solutions; (5) Reminder tools for everyday use.

### 3.1 Feeling fresh vs. feeling indifferent: a broad spectrum of attitudes

Participants' attitudes to oral health varied significantly and could be understood to cover a broad spectrum. Some experienced strong intrinsic motivation, with statements like “*I like dental hygiene. It just happens automatically,”* reflecting a sense of satisfaction. Habits also played a part, as one participant stated “*I have gotten into the habit of not being able to go out without brushing my teeth. I do it several times a day.”*

Interactions with dental practitioners were strongly connected to views on oral health. For some, the dentist's guidance was a source of comfort and assurance. One participant mentioned, “*The dentist tells me about a lot of things, which makes me feel safe.”* This kind of interaction could foster a sense of security and may motivate better oral hygiene practices. However, others were driven by fear or embarrassment and avoided visiting dental clinics altogether: “*I'm terrified of the dentist, so I'm not going.”*

Some participants considered oral health was out of their control due to genetic factors, for example one participant noted, “*I have bad genes, so I just get cavities all the time*.” A lack of intrinsic motivation was apparent in utterances like “*Nothing makes me do it unless mom nags for half an hour.”*

Expressions pointing at hopelessness and indifference towards oral health were also identified. For instance, a participant stated, “*I've started to not give a damn in life, everything that comes out of adults’ mouths is a bit like whatever.”* Some revealed how dismissive attitudes would extend to various aspects of life, including oral health which was not viewed as a high priority.

External factors also served as motivators to maintain good oral health habits, encompassing both positive incentives and pressure or coercion. While one participant recommended positive reinforcement, saying, “*Don't say it's homework, gives bad associations, but give them a gift, a toothbrush and some floss*,” others were motivated by the tangible repercussions of poor oral health, like high dental bills: “*Mom showed me the dental bill and it's very expensive, it's scary.”*

### 3.2 Bridging gaps, building habits: collaborative efforts in oral care

The participants viewed everyday oral health as a collaborative endeavor. They identified the importance of having a supportive ally that cares by providing guidance, feedback, and reassurance. Key people in typical everyday contexts were parents, siblings, other family members, caregivers, or motivating friends. These individuals significantly contributed to shaping the participants' oral health habits.

Some participants humorously recognized the necessity of parental reminders. As one participant mentioned, “*I have parents who are a pain in the ass and keep nagging.”* This statement, though expressed with a hint of frustration, underscores how consistent reminders can help enforce good oral health practices. The influence of mothers was particularly commonly noted. Drawing from personal experiences, parents can foster good habits. As one participant recalled, “*It's my dad, who says he himself has such ugly teeth and doesn’t want me to get the same as him.”* These past experiences can serve as potent reminders of the potential consequences of neglecting oral health. In addition to offering support, parents who are well-informed play a crucial role in laying the groundwork for success. As one participant expressed, “*Parents should be shown the consequences of not brushing their teeth, because not all adults know why it is important.*”

Beyond biological parents, various caregivers like foster parents play a key role in shaping oral health habits. As participants noted, the importance of good oral hygiene can differ among caregivers, highlighting the influence of diverse family structures. This extends to siblings, friends, and professional caregivers who can also contribute to promoting good oral health. As one participant pointed out, “*Not everyone has parents*.”

Despite best efforts, maintaining good oral health can be challenging due to factors like family dynamics. One participant mentioned that parental reminders can sometimes be ignored, “*They tried to get my mom to remind me to brush my teeth, but then it goes in one ear and out the other because it was something my mom told me,”* emphasizing how family environment and parent-child relationships can negatively impact the success of promoting good oral hygiene.

Participants also highlighted the critical role dental professionals could play in promoting oral health not just in clinics but in daily life as well. They suggested that dentists could actively motivate children to practice good oral hygiene. However, there was a noted shortfall in dental health education in schools, and several participants recommended dental professionals extend their influence by visiting schools to educate young people on oral health. As one participant put it, “*They are good at their job, but not so good at informing children and young people. [They should] visit the school and talk to children*.”

### 3.3 “Create good experiences at the dentist so people come back again”

The need for tailored communication and detailed information surrounding their dental visits to create positive experiences was emphasized. Before the visit or procedure itself, participants would like to know which procedures would take place, their purpose, and who would be performing them. One participant expressed, “*I want to know a bit about the dentist—who is going to be looking in my mouth?”* Clarity on their rights, such as who could accompany them and, using stop signs for discomfort, whether the procedure would be painful, and potential treatment costs, was considered important. The participants suggested that clinics should provide anticipatory information about the dental visit and a summary afterwards. Some participants also wished to have a say in scheduling their next appointment, promoting a collaborative approach to their dental care.

Once at the clinic, the quality of communication with all dental personnel was considered crucial. Participants valued dental practitioners who were calm, patient, and uplifting, stating preferences like “*create good experiences at the dentist so people come back again”* and “*try to give us hope instead of pushing us down.”* The participants appreciated dental professionals who take the time to motivate and make recommendations. They appreciated marked opportunities for active engagement, such as choosing background music or using a stop signal. Effective communication and consent before physical touch were considered essential for building trust and comfort. As one participant noted, professionals should “*ask if it's okay before they touch you.”*

In addition, rewards played an important role in rounding off the dental visit. The reward system, which in a Norwegian dental clinic context is often associated with concluding the appointment, was frequently discussed as an important marker. For many participants, it served as an essential motivator and contributed to a positive dental visit experience, as illustrated by statements like: “*Everyone should get a reward, regardless of age”* and “*Some may need a reward in advance also because it can have a reassuring effect.”*

Lastly, participants expressed that the dental clinic's ambiance and aesthetics played a significant role in shaping their overall experience. They proposed a multi-faceted approach to make the environment more welcoming. Visually, they suggested the addition of ceiling-mounted entertainment, like films, as a helpful distraction during procedures. They also favored a more inviting waiting room with soothing colors, natural elements like plants and avoidance of intimidating imagery in favor of fun, educational cartoons. Moreover, some participants suggested playing youth-friendly music to enhance relaxation and personalize the patient experience.

### 3.4 Requested qualities in oral health promoting solutions

Participants preferred oral health promotion solutions with various qualities. This theme highlights six central attributes derived from their suggestions.

#### 3.4.1 Cheerful aesthetics

The participants leaned towards colorful visuals, engaging illustrations, and unique, amusing concepts. One participant humorously suggested, “*If Hello Kitty held dental floss in one hand and a gun in the other, then I would have laughed every time I saw it,”* underscoring the appeal of lighthearted and funny aesthetics. Cartoons, even for teenagers, and temporary tattoos with oral health messages were also welcomed.

#### 3.4.2 Simplicity and affordability

Solutions should be easy to use and low cost, with examples such as step-by-step instructions, demonstrations, and simple to-do lists. Tangible reminders that could be refreshed twice daily were favored, along with easily accessible standard information.

#### 3.4.3 Credibility

Information should come from a trustworthy, professional source to inspire trust and confidence in users.

#### 3.4.4 Timely notifications

Participants valued reminders, such as those for upcoming dental appointments or tailored push notifications, to keep them on track.

#### 3.4.5 Pedagogical and relational aspects

Participants emphasized the significance of clear, straightforward information. Some participants noted the need for explanations, while others favored a more consequential approach, suggesting that “*intimidation works. You don't want to walk around with ugly teeth.”* Other participants advocated for a balanced approach, incorporating elements of humor and positivity.

#### 3.4.6 Preferences in written and visual communication

There were mixed feelings about the use of emojis and the anthropomorphizing of objects, like dental floss. However, participants agreed that messages should be motivating, affirming, and not overly bossy. The messages should be personalized and conveyed in a kind and simple manner.

### 3.5 Reminder tools for everyday use

Routines, reminders, appointments, and regularity were revealed as important components for building good dental health habits. This theme comprises suggestions for reminder tools to promote oral health in everyday life.

The participants envisioned using both digital and physical resources to encourage and remind people to maintain their oral hygiene. They suggested using digital reminders such as a personalized app with custom alarms, tooth-brushing games, and a fun tooth-brushing song that could also serve as an alarm. In addition, some participants proposed using an app to keep track of dental check-ups and to provide personalized tips based on the last dental visit. Other participants proposed the idea of digital demonstrations, for instance, suggesting the use of “*a series of pictures, or film, ‘This is how you brush your teeth, this long you should keep the water in your mouth'”* to provide clear and easily accessible instructions. Participants appreciated the idea of receiving messages or calls as a check-up on their oral hygiene progress.

Physical reminders were also popular. Many participants liked the thought of step-by-step instructions on paper or in brochures. They wanted reminder notes, illustrated flyers, and posters to keep around the house. Some wanted lists on the bathroom mirror. Physical reminders like stickers or tattoos could both remind and reward. Some participants suggested color-changing tools or calendars to track progress.

Creating triggers and incentives was another idea. Several participants suggested acronyms, keywords, or songs to make routines enjoyable. They mentioned powerful visuals like those on cigarette packs to remind them of the consequences of neglecting oral health. Making routines fun included using flavored toothpaste, cool toothbrushes, or funny images.

## 4 Discussion

Based on the results, possible implications for providing tailored oral health promotion in adolescents are discussed. Furthermore, we consider how the findings may relate to social inequalities.

### 4.1 Reflections on the key results

The five presented themes comprise adolescents' views on oral health care and promotion in a Norwegian context. The themes, which partially overlap, provide important value for improving services and interventions in terms of oral health promotion.

The participants had a broad range of emotions associated with oral health, from satisfaction and safety to shame and fear, as captured by the theme “*Feeling fresh vs. feeling indifferent:* A broad spectrum of attitudes*.”* The theme emphasizes a variety in attitudes and motivations towards own oral health, along with everyday practices and resources utilized to maintain it. Attitudes and situations related to oral health are not static and can indeed be changed. For some, oral health care becomes a priority only when they are in the right frame of mind, resulting in inconsistent care. Good insight into one's oral hygiene practices does not necessarily make a young person resistant to changing those habits, rather the significance of dental hygiene can be obscured in certain environments or family situations, leading to a lack of prioritization of oral care. As a foundation in oral health promotive work, it is crucial to understand that a variety of factors influence the way we form and maintain health habits. Therefore, there is a clear need for tailored strategies to effectively promote oral health practices among young people, as also confirmed by other studies (7, 29). Emotional and motivational aspects of oral health are critical in shaping healthy habits and serve as an underlying layer to the other identified themes.

The critical role of supportive allies in oral health promotive work is thematized in “*Bridging gaps, building habits: Collaborative efforts in oral care.”* Importantly, the participants recognize that learning about and maintaining healthy habits depends on collaboration. The participants' statements highlighted several factors that can hinder successful collaboration in oral care. These include non-traditional family structures, challenges related to family dynamics, and individual attitudes toward oral care. This underscores the importance of a comprehensive approach to oral health promotion. The participants' suggestions included bridging potential gaps through a strengthened collaboration between homes, dental professionals, and educational institutions. Questions about oral health education, such as in a school context, were not specifically included in the tooth assignment. However, participants raised this as a suggestion for future oral health promotion, in response to some of the questions. For oral health promotion, it appears beneficial to identify the relevant ecosystems that could help adolescents foster healthy habits. As constructive and detrimental habits are often inherited from one's environment, the importance of collaboration to encourage positive oral health habits should guide the development of services and interventions.

Regarding the setting of dental clinics, the importance of supportive relationships in oral care is also underscored in the theme “*Create good experiences at the dentist so people come back again.”* Interestingly, most participants focused on the responsibilities and conduct of dental health personnel in interactions with patients, rather than on their own behaviors. The participants offered recommendations for creating good experiences, focusing on tailored communication and actionable suggestions for improvement. Key points such as pre-visit information, clear communication, personal agency, transparency about cost, and positive reinforcement through rewards were covered. Hence, visiting the dental clinic is far from limited to treating a dental issue, but also about nurturing a sense of comfort and trust, which could encourage an ongoing commitment to oral health. The participants emphasized creating an environment and experience that inspired patients to maintain regular check-ups and ensure optimal oral health. The participants' suggestions correspond to a comprehensive vision for a dental clinic that is not only functional but also engaging and comforting, addressing both visual and auditory aspects to ease potential patient anxiety and encourage ongoing engagement as well as willingness to return for future visits (30). The importance of honesty, humor, and respect in fostering a positive patient-dentist relationship was also emphasized, extending to broader contexts like school visits. These elements collectively influence patient engagement and as such their attitudes toward dental health.

The two final themes, “*Requested qualities in oral health promoting solutions”* and “*Reminder tools for everyday use”,* capture a broad array of qualities that participants considered important for oral health promotion and, more specifically, for reminding and motivating adolescents about oral health practices. Central qualities included aesthetics, simplicity, credibility, effectiveness, pedagogical aspects, and communicative preferences, as is also shown in other studies, e.g., (31). Preferred sources included various suggestions for both digital and physical resources. In addition, creative triggers like acronyms, keywords, or songs, and powerful visual reminders were suggested. Moreover, the value of using positive motivation over scare tactics dominated the participants' recommendations. While some participants did indicate that they would be motivated by pressure or obligation, these approaches can also be viewed as coercive rather than encouraging. As a result, they may generate negative emotions surrounding oral health practices, even if they could be regarded effective in some cases.

The limited discussion about diet and sugary drinks during conversations with the participants was somewhat unexpected but may be attributed to several factors. One possible reason is that adolescents may not fully comprehend the impact of their diet on oral health, leading them to downplay its significance in the discussions and rather place greater emphasis on issues that are more visibly associated with their oral health. Another reason could be that participants might feel discomfort or embarrassment when discussing their dietary habits or might perceive the dental consultation as primarily focused on their current dental condition and treatment, causing them to prioritize discussions about their teeth and oral care routines rather than their dietary choices.

### 4.2 Reflections on the theme of social inequalities

Social inequalities represent a major public challenge within oral health, hitting deprived areas in both industrialized and non-industrialized countries alike (32). Unequal distribution of resources—such as wealth, income, education, family background, and power—leads to disadvantages in health and quality of life for individuals, families, and societies. Regarding oral health outcomes in children and adolescents, social capital has been shown to be an important factor benefiting health and, hence, useful for planning public health strategies (33). The concept of social capital can be understood as collective resources that emerge within prevalent social networks or in societal structures marked by mutual trust (34). The visible nature of teeth makes oral health a clear social marker, across countries with different levels of socio-economic status and welfare systems.

The theme, “*Bridging gaps, building habits: Collaborative efforts in oral care,”* confirms how support systems like family and educational institutions can act as social capital, making oral health care more topical and accessible. The critical role of social capital becomes even more apparent when considering the emotional and motivational spectrum of oral health attitudes. A rich reservoir of social capital in families typically results in prioritizing oral health, instilling a positive attitude toward it. On the flip side, an absence of such capital usually translates to oral health negligence, leading to a cycle of poor practices and indifference (35).

While social capital plays a role, income and educational levels also significantly shape oral health outcomes. In the Norwegian context, where dental care is free of charge for the target group, it is intriguing to note that cost remains a concern for some participants. This suggests that the issue is more nuanced and hints at two potential issues. Firstly, there might be a general lack of awareness that certain age groups are entitled to free dental care. This gap could result from ineffective communication from health services or cultural and linguistic barriers. Secondly, even in a system designed to be accessible, the hidden or indirect costs can still pose challenges for lower-income families. These costs can range from travel to the dental clinic to taking time off work, and they can create a financial burden that hinders optimal oral health practices. Lack of clear communication about these costs can intensify the problem, leading to apprehensions and misunderstandings. Therefore, while education and income continue to be significant factors contributing to health disparities, the unexpected concern about cost highlights the complex ways in which societal factors can influence oral health (36).

Language inclusivity is another dimension worth emphasizing. First, the participants highlighted the importance of using culturally sensitive language in oral health communication, pointing at youth culture. A second, related point is that individuals who are not fluent in a community's dominant language may hesitate to seek dental services. This could be due to a lack of accessible and understandable information on oral health. Such barriers can be particularly pronounced in areas with significant immigrant populations, underscoring the need for language-sensitive health promotion work (37).

Furthermore, it is vital to acknowledge the educational gaps among parents. Some parents might be unaware of the significance of oral health due to educational disparities in their upbringing. Educating parents about oral health and the long-term consequences of oral health neglect could empower them to guide their children more effectively. By rolling out targeted awareness campaigns that spell out the long-term health and financial implications of oral neglect, parents can be better equipped to guide their children (38). A relevant study is that of Nanjappa et al. (36) presenting a tool that facilitates effective interaction between dental health support workers and families facing socioeconomic challenges.

Lastly, it is important to consider individuals with specific needs, including those diagnosed with conditions like dyslexia, ADHD, or cognitive impairments. These populations encounter unique challenges in accessing and maintaining oral health. Customized interventions, like multi-sensory instructional methods or simplified guidelines, could make a difference. Also, equipping dental healthcare providers with training to cater to these needs can prove instrumental (39). Although participants did not specifically mention these conditions, they strongly suggested the benefits of providing repeated messages and visual support. These are well known means for increased patient attention, comprehension, recall and adherence in health communication research (40).

## 5 Strengths and limitations of the study

Recent research has emphasized the need for a more nuanced, targeted approach to improve adolescent oral health in Norway, and our study provides more details into what participants from the target group experience and suggest for design of adolescent-focused promotive and preventative solutions in a Norwegian context. The results show how adolescents experience varying attitudes and needs concerning oral health and emphasize the importance of personalized, collaborative approaches. This can be useful for understanding and planning new models for educating adolescents on oral health care. Moreover, aspects that contribute to create positive experiences with dental practitioners, in and outside the dental clinics, are revealed. In addition, qualities that are considered important in health-promoting tools crucial for daily oral habits have been identified. These findings should be useful for improving oral health services and oral health promotion in a Norwegian and Nordic context and are also expected to be relevant for dental professionals and public health organizations in wider international communities with similar welfare systems. Adolescence represents a critical phase of transition in many aspects, and looking specifically at how health services can acknowledge the value of co-creation to offer care that is more tailored applies to many geographical contexts and healthcare systems. Moreover, social inequalities represent a global challenge and our reflections of how the results can be viewed considering social capital could hopefully serve as an inspiration for future studies.

A limitation of the study is that the authors were not present in collecting data. Moreover, details about the participants' backgrounds were not collected. Aspects such as gender differences and residence situation might have significance. The number of responses for and against a specific issue were also not counted. This may affect the conclusiveness of the findings. However, a strength lies in the applied feedback loops in which representatives of the participants actively participated in formulating questions, presenting key points to the project group, and had access to preliminary findings presented both orally and in writing.

As is the case with focus group discussions, conversation flow influences participants' perspectives. A strength can be shared engagement, whereas a limitation can be that some aspects are difficult to raise, like for example the topic of diet which was not a topic brought forward in the data material.

## 6 Conclusion

Our study sheds light on the perspectives of adolescents on oral health care in Norway, revealing both challenges and opportunities for improvement. Five central themes were identified. These capture adolescents' experiences and desires for oral health promotion, from individual attitudes to dental care experiences shaped by social interactions. The findings suggest that tailored, collaborative approaches could enhance the success of oral health interventions for this age group. We also offer reflections on the theme of social inequalities in oral health care access and literacy. By considering socioeconomic disparities, a long-term research aim is to contribute to a more equitable oral health care system in Norway and potentially in similar international settings.

## Data Availability

Anonymized transcripts and notes that support the findings of this study are available from the corresponding author, upon reasonable request.
